# Acute Gastric Hemorrhage due to Gastric Cancer Eroding Into a Splenic Artery Pseudoaneurysm: Two Dangerously Rare Etiologies of Upper Gastrointestinal Bleeding

**DOI:** 10.7759/cureus.10685

**Published:** 2020-09-27

**Authors:** Anabel Liyen Cartelle, Pearl Princess Uy, John Erikson L Yap

**Affiliations:** 1 Gastroenterology and Hepatology, Medical College of Georgia at Augusta University, Augusta, USA

**Keywords:** pseudoaneurysm of splenic artery, gastric tumor, upper gastrointestinal bleed

## Abstract

Splenic artery pseudoaneurysms (SAPs) are rare causes of upper gastrointestinal bleeding (UGIB), with less than 250 reported cases in the literature. The highest incidence of SAPs is in patients with a history of acute or chronic pancreatitis or splenic artery trauma. SAP in the setting of gastric malignancy is an exceedingly rare finding. We present the unusual hospital course of an 82-year-old male with advanced gastric cancer presenting with UGIB secondary to a visceral communication between his known gastric malignancy and a SAP.

## Introduction

An upper gastrointestinal bleeding (UGIB) is defined as any bleed that originates proximal to the ligament of Treitz. Acute UGIB is a common occurrence, affecting on average 100-200 individuals per 100,000 annually [1]. Although most episodes resolve on their own, they result in significant morbidity and mortality and incur an estimated health care cost of $2 billion/year [1,2]. Common sources of UGIB include peptic ulcer disease (PUD), esophageal varices/ulcers, Dieulafoy lesions, arteriovenous malformations, gastritis/duodenitis, and Mallory-Weiss tears [2]. Gastric malignancies account for a very small fraction of all UGIB events, about 2-8% of all cases [3]. Even rarer still are UGIB secondary to ruptured visceral aneurysms or pseudoaneurysms. Unlike splenic artery aneurysms (SAAs), splenic artery pseudoaneurysms (SAPs) are often symptomatic on presentation, with patients exhibiting abdominal pain and signs of UGIB including melena or hematochezia [4,5]. Despite their rarity, timely detection of ruptured SAPs is critical to the survival of patients as mortality can reach 90% in untreated cases [6]. We present the unusual hospital course of an 82-year-old male with advanced gastric cancer presenting with UGIB secondary to a visceral communication between his known gastric malignancy and a SAP.

## Case presentation

An 82-year-old male recently diagnosed with a gastric malignancy at an outside institution presented to the emergency department as a transfer after a one-day history of worsening weakness and dyspnea on exertion. He had been having intermittent hematemesis for the past month and last vomited the day prior to presentation. He also reported melena and unintentional weight loss, but no abdominal pain or distention. He admitted to chronic ibuprofen use averaging 400 mg a day, but he denied any history of tobacco use, alcohol abuse, or recreational drug use. He had no previous abdominal surgeries and denied any known family history of gastrointestinal (GI) cancers.

On arrival to the ED the patient was afebrile, tachycardic in the low 100s, and hypotensive at 102/40 mm Hg. He was pale and had dry mucous membranes on examination. His abdomen was nontender and nondistended, but he had melena on digital rectal examination. Initial labs were significant for a hemoglobin of 7.9 g/dL, platelet count of 232,000/mm^3^, international normalized ratio of (INR) of 1.2, blood urea nitrogen (BUN) of 21 mg/dL, and creatinine of 0.87 mg/dL. All other values were within normal limits. Chest X-ray was unremarkable. While in the ED, he had an episode of worsening hypotension following a bowel movement, but improved following IV fluid resuscitation and a transfusion of packed red blood cells. IV proton pump inhibitors were administered, and the patient was eventually stabilized enough to warrant admission to the step-down unit.

**Figure 1:**
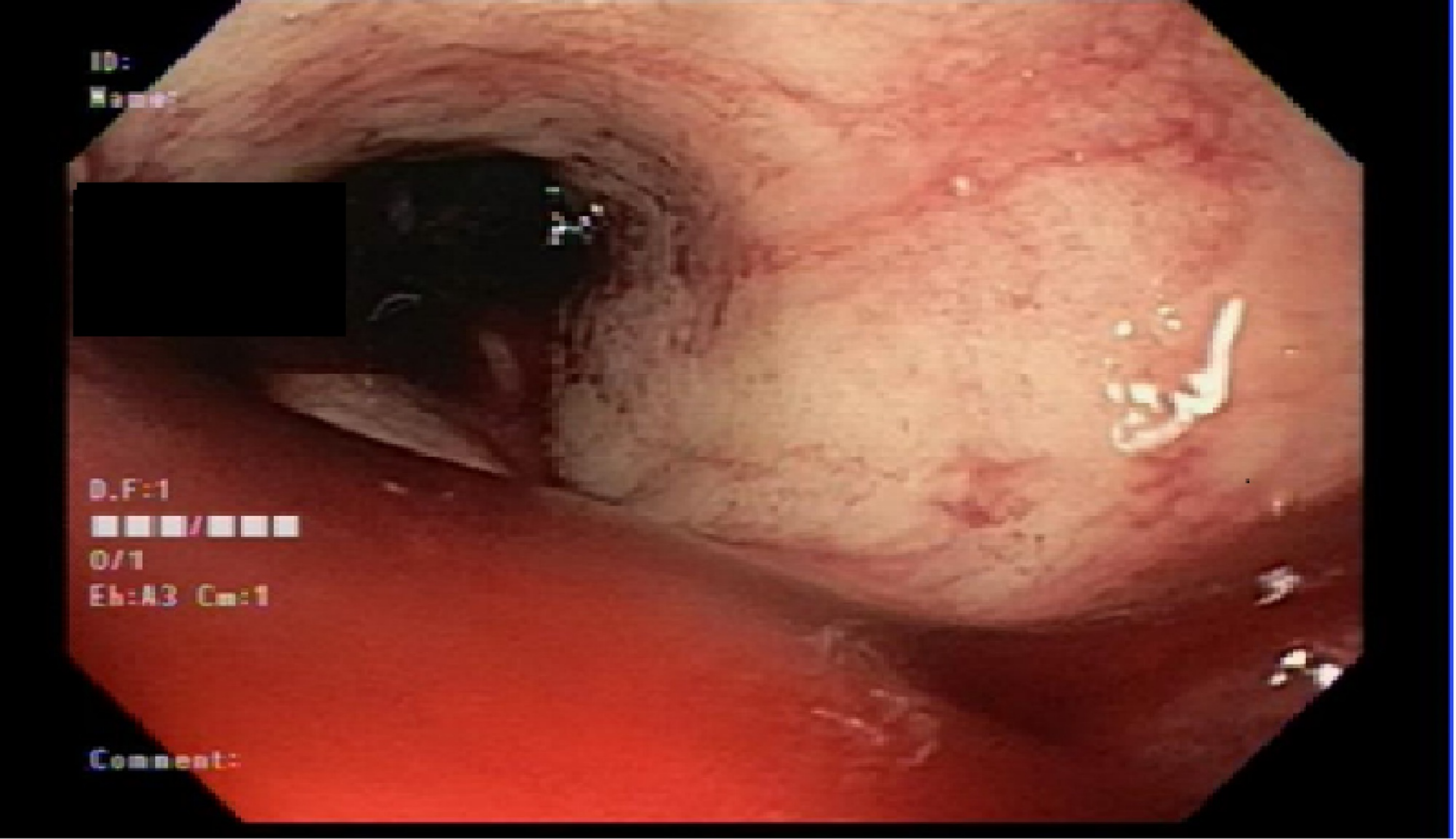
EGD: large amount of blood and clots seen at the gastric lumen. The source of bleeding could not be visualized in spite of copious irrigation and suction. EGD, esophagogastroduodenoscopy

The GI service was consulted and an esophagogastroduodenoscopy was performed, which revealed large amounts of bright red blood with clots in the gastric lumen obscuring adequate visualization of the mucosa (Figure 1). Despite copious irrigation and aggressive suctioning, it was impossible to localize the bleeding source or attempt any endoscopic intervention. Additionally, the patient began to deteriorate during the procedure and had to be intubated. Massive transfusion protocol was initiated, and given the presence of active bleeding, the interventional radiology (IR) service was consulted for an emergent angiography with possible embolization.

**Figure 2:**
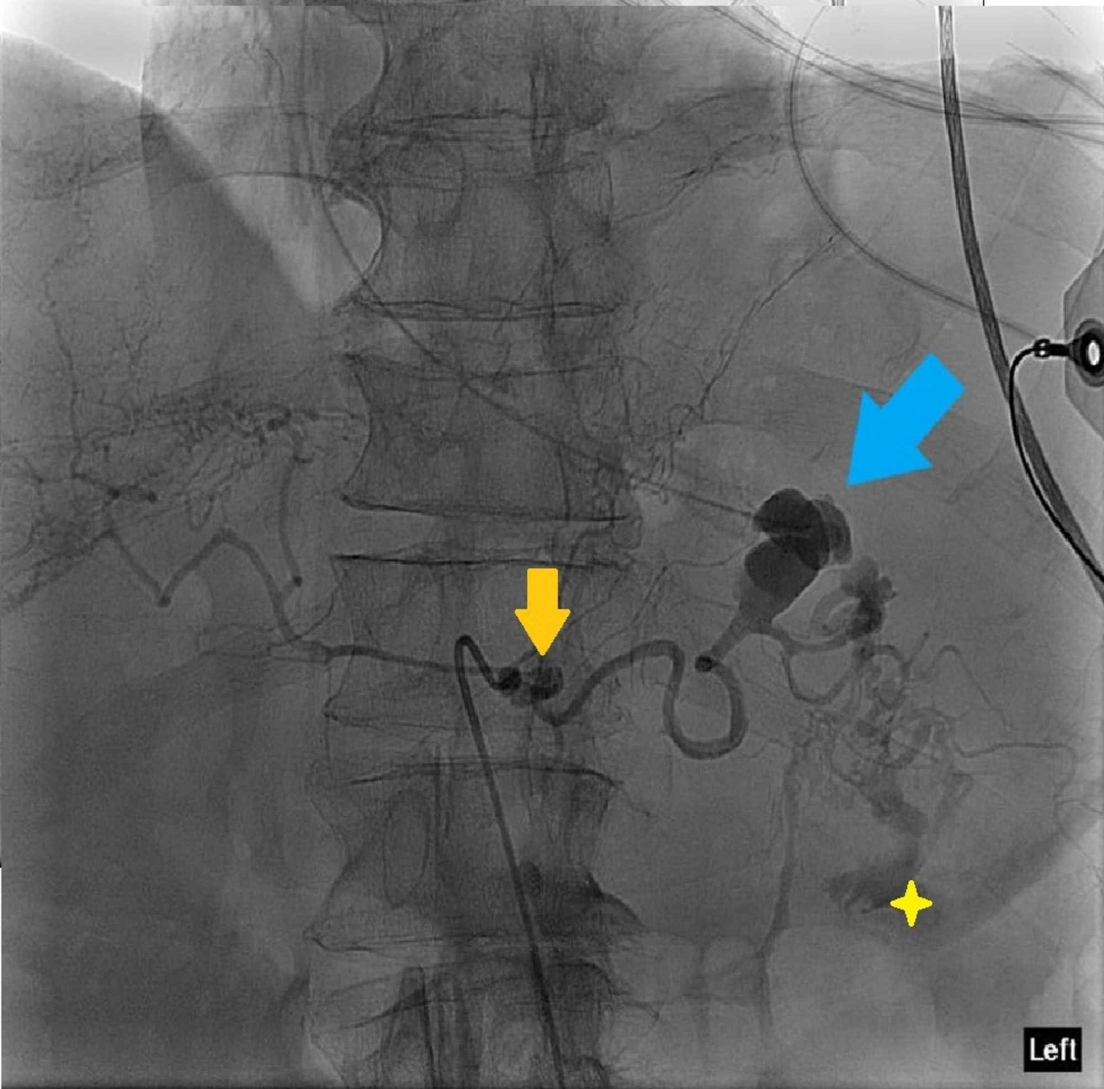
Angiography of the celiac axis demonstrated a large distal splenic artery pseudoaneurysm near the splenic hilum (blue arrow) with large volume active bleeding into the gastric lumen (yellow star) likely due to arterial erosion from a known gastric tumor. An additional small aneurysm was noted in the celiac artery without active bleeding (orange arrow).

Angiography of the celiac axis demonstrated a large distal SAP near the splenic hilum with large volume of active bleeding into the gastric lumen due to arterial erosion from the known gastric cancer. An additional small aneurysm was noted in the celiac artery without active bleeding (Figure 2). Multiple attempts were unsuccessful in traversing past the proximal celiac axis aneurysm to embolize the more distal bleeding SAP. At this point, emergent surgical consultation was taken, but the patient became increasingly unstable and eventually succumbed to cardiac arrest despite aggressive resuscitation efforts.

## Discussion

SAPs pose a significant diagnostic challenge for clinicians. From the limited case reports and case series available, the formation of these lesions has been largely attributed to inflammatory damage secondary to acute or chronic pancreatitis (52%), surgical/blunt trauma (3%/29%), and, to a much lesser extent, PUD (2%) [7]. In contrast to true aneurysms, which involve all three layers of the vessel - intima, media, and adventitia - pseudoaneurysms typically consist of the intima and media only. Like SAAs, SAPs are the most common types of visceral pseudoaneurysm encountered. Unlike SAAs, SAPs are almost always symptomatic on presentation and carry a much higher risk of rupture (37-47% vs. 2-3%) [8]. Acute GI hemorrhage evidenced by physical examination findings of melena and hematochezia is often attributed to bleeding into the pancreatic duct, a phenomenon known as hemosuccus pancreaticus [9]. However, cases have also been reported of acute pseudoaneurysm bleeding into the stomach, colon, and the peritoneal cavity directly [9,10]. Given their rarity with less than 250 reported cases, the timely detection of ruptured SAPs in the setting of an acute UGIB requires a very high level of suspicion. In our patient’s case, there was no contributory past medical history that would have pointed us in the direction of an SAP. Additionally, the generation of a broader differential to explain the UGIB was hindered by the presence of his gastric cancer, another condition that also explained the sudden blood loss.

While primary bleeding from gastric cancer accounts for a very small percentage of all UGIB, these patients can also bleed from concomitant benign causes such as peptic ulcers, esophageal and gastric varices, hemorrhagic gastritis, and angiodysplasia [11]. Based on review of current English literature, there has only been one other reported case of a patient presenting with a UGIB secondary to a ruptured SAP in the setting of an underlying gastric cancer [12]. In traditional bleeding gastric cancers, therapeutic intervention is usually prioritized in the following order: first endoscopic treatment, second transcatheter arterial embolization, and third emergent gastrectomy [13]. Although upper endoscopy has a high reported success rate in treating actively bleeding inoperable gastric cancers, its therapeutic utility is severely limited by profuse bleeding [11].

For UGIB secondary to ruptured SAPs, multiple case reports have described a failure to identify the bleeding source through endoscopy either due to (1) poor visualization as a result of profuse bleeding or (2) a lack of visible intraluminal abnormalities [7,14]. In these cases, detection of the pseudoaneurysm was picked up on subsequent computed tomography (CT). In the event of endoscopic failure to identify a source of the bleeding, the American College of Radiology guidelines recommend either a catheter angiography or CT angiography (CTA) for evaluation [15]. For hemodynamically stable patients, CTA has a reported sensitivity of up to 94.7% and a specificity of 90.0% in detecting anomalies within these splanchnic vessels [16]. Both true aneurysms and pseudoaneurysms present with very similarly on CTA as arterial phase enhancing outpouchings from the vessel wall. However, pseudoaneurysms tend to have more irregular margins and are usually surrounded with hematoma [17].

Once detected, intervention is considered necessary for all ruptured and unruptured visceral pseudoaneurysms, ruptured visceral aneurysms, and unruptured visceral aneurysms measuring >2 cm in diameter [18]. Depending on the hemodynamic stability of the patient, endovascular embolization is often the first-line therapy. Techniques such as the “sandwich” method, which involves placing coils proximal and distal to the lesion, have shown success in stopping acute bleeding and preventing collateral driven rebleeding. Alternatively, for lesions with narrow outpouching necks, the packing of the vascular sac with coils can also be employed without requiring complete vessel occlusion [19]. Success rates in the treatment of SAP/SAA using embolization have been reported to range between 75% and 98% [20]. It has been recognized that higher failure rates are observed in lesions that are in torturous arteries, have wider necks, or are more distally located, such as in our patient’s case [20]. Surgical repair or resection of the aneurysm without splenectomy is typically considered second-line treatment especially in a hospital center with advanced vascular surgery or IR service. Avoidance of splenectomy is prioritized as it carries a long-term risk of bacterial infections [20]. Overall, surgical intervention carries a morbidity and mortality risk of 9% and 1.3%, respectively [8], with emergent procedures in hemodynamically unstable patients escalating to higher percentages.

## Conclusions

SAPs are very rare vascular lesions with less than 250 reported cases in the English literature. Although patients usually present with abdominal pain and signs of overt UGIB, evaluation for a ruptured SAP remains a diagnostic challenge and requires a very high level of suspicion. Clinicians must deduce the probability of occurrence through evaluation of the patient’s history of pancreatitis or blunt force or surgical trauma to the spleen and its vasculature. There is an additional level of complexity if the patient has a pre-existing condition that could also explain UGIB, such as a gastric malignancy. Regardless, if endoscopic intervention fails to locate the source of bleeding, CTA or angiography is the preferred imaging modality to evaluate for splanchnic vasculature complications. Endovascular embolization remains the preferred primary intervention, with open surgery left as the last resort. This case highlights the importance of prompt diagnosis and treatment as well as consideration of SAP as one of the differentials of upper GI bleed. To the best of our knowledge, the case we have presented is the second reported occurrence of a UGIB caused by a ruptured SAP in the setting of gastric cancer.
